# Risk factors for saddle-related skin lesions on elephants used in the tourism industry in Thailand

**DOI:** 10.1186/s12917-015-0438-1

**Published:** 2015-05-19

**Authors:** Scarlett Magda, Olivia Spohn, Taweepoke Angkawanish, Dale A. Smith, David L. Pearl

**Affiliations:** Department of Pathobiology, Ontario Veterinary College, University of Guelph, Guelph, ON N1G 2W1 Canada; Department of Population Medicine, Ontario Veterinary College, University of Guelph, Guelph, ON N1G 2W1 Canada; The National Elephant Institute, Hangchart, Lampang, Thailand

**Keywords:** Elephants, Saddle sores, Thailand, Tourism, Multi-level modelling

## Abstract

**Background:**

Lesions related to working conditions and improper saddle design are a concern for a variety of working animals including elephants. The objectives of the present study were to determine the prevalence of cutaneous lesions in anatomic regions (i.e., neck, girth, back, tail) in contact with saddle-related equipment among elephants in Thailand working in the tourism industry, and to identify potential risk factors associated with these lesions. Data for this cross-sectional study were collected between May 2007 and July 2007 on 194 elephants from 18 tourism camps across Thailand.

**Results:**

There was a high prevalence (64.4 %; 95 % CI 57.3 – 71.2) of active lesions, most often located on the back region. Using multilevel multivariable logistic regression modelling containing a random intercept for camp we identified the following risk factors: increasing elephant age, the use of rice sacks as padding material in contact with the skin, and the provision of a break for the elephants. Working hours had a quadratic relationship with the log odds of an active lesion where the probability of an active lesion initially increased with the number of working hours per day and then declined possibly reflecting a “healthy worker” bias where only animals without lesions continue to be able to work these longer hours.

**Conclusions:**

While we recognize that the cross-sectional nature of the study posed some inferential limitations, our results offer several potential intervention points for the prevention of these lesions. Specifically, we recommend the following until longitudinal studies can be conducted: increased monitoring of older elephants and the back region of all elephants, working less than 6 hours per day, and the avoidance of rice sacks as padding material in contact with skin.

**Electronic supplementary material:**

The online version of this article (doi:10.1186/s12917-015-0438-1) contains supplementary material, which is available to authorized users.

## Background

The Asian elephant (*Elephas maximus*) has been captive in Thailand for approximately 4000 years, and is classified as a working animal under the Beast of Burden Act of 1939 [1–5]. Although traditionally used in war and religious ceremonies, in the 18th and 19th centuries, the Asian elephant was used as a source of transportation for logs and people, primarily in the logging industry [1, 3–6]. However, over the past century there has been a substantial decline in the population of working elephants, from approximately 100 000 to fewer than 3 000 elephants today [2, 4, 5, 7]. This decline was due to an increase in the use of machinery instead of elephants, in conjunction with the logging ban of 1989; thus, the need to capture wild elephants as replacement animals was significantly reduced [1, 3–7]. The remaining elephants are now primarily found working in the tourism industry, with 135 camps identified across Thailand in 2011 by the National Elephant Institute [1,3-7; T. Angkawanish, personal communication]. Although tourism offers a limited solution, many researchers are in agreement that the overall welfare of these elephants depends greatly on their economic value, utility, and the financial position of their owners; all of which are considerably enhanced as a result of the tourism industry [1–5]. Concerns over poor welfare due to factors such as artificially created social groups, inadequate quantity and quality of food, and poor veterinary care have been investigated to some extent [4, 5, 8–12].

However, relatively few studies have investigated the physical effects of work on this population of working elephants. There is only a brief mention in one report of the presence of abrasions caused by the movement of the tourism saddle and girth strap [13]. This is in contrast to the domestic equid industry involving donkeys, horses and mules, where a number of studies have implicated overworking/overloading and improper saddle/harness design as major causal factors for external lesions [14–18]. Similar welfare concerns are likely relevant to working elephants. Consequently, the objectives of this study were to determine the nature of equipment-related skin injuries in a population of Asian elephants working in the tourism industry by determining the following: 1) the prevalence of external injuries in four saddle related areas: neck, girth, back and tail, among working tourism elephants in Thailand; and 2) potential risk factors for skin lesions related to working conditions, including demographic factors and saddle design.

## Methods

Data for this cross-sectional study were collected between May and July 2007, by the lead researcher (S. Magda) who worked in partnership with The Elephant Hospital of the National Elephant Institute, formerly known as the Thai Elephant Conservation Centre. Data were collected from 18 camps across Thailand that received routine visitation from veterinarians from the Livestock and Wildlife Hospital of Mahidol University, or the National Elephant Institute’s Elephant hospital. Elephants were eligible for the study if they worked in the tourism industry and wore a saddle (n = 194). Each elephant underwent a veterinary examination and elephant keepers (mahouts) answered a series of standardized questions pertaining to each elephant and its saddle-wearing history using a translator (Additional file 1).

The outcome of interest for the present study was the presence of an ‘active lesion’ in any one of four anatomical areas: neck, girth, back and tail. Lesions were placed initially into 10 categories (with category ‘0’ being no lesion). However, to achieve adequate power for statistical analyses, lesions were classified as either ‘active’ or ‘inactive/absent’. Active lesions were those that could be described as rubbed/pink, raw, a full depth ulcer, an abscess (closed or draining), a healing lesion, or depigmented skin. A body region was categorized as having an inactive/absent lesion if there was no lesion, a healed lesion/scar, or a callus.

The following independent variables were investigated to determine their association with the presence of an active lesion: age of the elephant (in years); region of the body (neck, girth, back, & tail); number of hours worked per day (defined as a period of time when the elephant was either carrying a tourist, or tethered with the saddle mounted and straps tied waiting to work); the provision of a break (defined as a period of time when either the girth strap was loosened, or the entire saddle was removed); weight of the saddle (light < 10 kg, heavy >10 kg); and contact material (the type of saddle pad material in contact with skin, including: shredded bark, bark, carpet, blanket, & rice sack). To allow for a greater number of observations per category, shredded bark and bark were combined into a single “bark” category.

Descriptive analyses were carried out using Microsoft Office Excel, 2010 (Microsoft Corporation, Redmond, Washington, USA) and STATA MP12.1 (STATA Corporation, College Station, Texas, USA). The prevalence proportions of elephants with at least one lesion (active or inactive), with at least one active lesion, and with no lesions were estimated. The prevalence of active lesions was also summarized by body region. Among camps, the prevalence of elephants with at least one active lesion was estimated. Explanatory variables of interest were summarized and tabulated by camp.

Using STATA MP12.1, univariable and multivariable multilevel logistic regression models were built to examine the association between the presence of an active lesion at any of the four anatomical sites (neck, girth, back, tail) and the explanatory variables: age, working hours, contact material, break, and saddle weight. These models contained random intercepts for camp and elephant to account for clustering, as multiple elephants were observed per camp, and multiple body regions were examined per elephant. The models were used to determine variance estimates at both the elephant and camp level. Decisions to include random intercepts were made based on model fit using Akaike’s Information Criterion (AIC) score, in combination with a likelihood-ratio test (statistical significance indicated by α ≤ 0.05).

Correlation among independent variables was tested using Spearman’s rank correlations. Any two variables found to be highly correlated at r ≥ 0.7 were assessed for completeness of data and biological relevance. This information was used to determine the most reliable variable to be included in the model, to avoid issues associated with collinearity. The linearity assumption was examined for continuous explanatory variables (age and working hours) using locally weighted regression with the lowess command. If the lowess smoother indicated a curvilinear relationship, a quadratic term was investigated and remained in the model if α ≤ 0.05. Variables with P ≤ 0.2 on univariable analysis (or that had ≥ 90 % of data collected) were considered for inclusion in the multivariable model. Variables included in the final multilevel multivariable model showed either statistical significance (α ≤ 0.05), or evidence of acting as a confounding variable (20 % or greater change to other statistically significant model coefficients when removed). Interaction effects were initially examined, but due to estimation issues associated with relatively small sample size, we limited our analysis to a main effects model. Pearson residuals and best linear unbiased predictors (BLUPs) were examined to assess the fit of the multilevel multivariable model.

## Results

### Descriptive statistics

In total, 776 body areas, from 194 elephants and 18 camps were examined for saddle related lesions. The prevalences of elephants (n = 194) with at least one active lesion, at least one lesion (active or inactive), and no lesions were found to be 64.4 % (95 % CI 57.3 – 71.2), 82.5 % (95 % CI 76.4 – 87.5), and 17.5 % (95 % CI 12.5 – 23.6), respectively. The distribution of active lesions by body area was as follows: 1.68 % on the neck (95 % CI 0.35 – 4.82), 30.73 % on the girth (95 % CI 24.06 – 38.04), 50.28 % on the back (95 % CI 42.72 – 57.82), and 17.32 % on the tail (95 % CI 12.08 – 23.67). Among camps, the average prevalence of elephants with at least one active lesion was 62.3 % (95 % CI 44.7 – 79.9), although this value ranged from 0 to 100 % (Table 1). The median value of prevalence of elephants with at least one active lesion by camp was 75.0 %. The number of elephants per camp ranged from 3 to 32 (Table 1). The age of the elephants in years ranged from 6 to 60, with an average age of 32.2 years. Scheduled working hours (i.e., the maximum number of hours of possible work per day depending on tourist volume) were found to be relatively consistent among elephants within a camp, although this value ranged between 2 to 10 hours among camps (Table 2). Whether or not the elephants were provided a break was also found to be a camp level variable (Table 2). Saddle weight was found to be equivalent for all elephants among the same camp, with 50 % of the camps using heavy (>10 kg) saddles, and 50 % using light (<10 kg) saddles (Table 2). The contact material of the saddle pad was found to vary both among camps, and between elephants within the same camp (Table 2).

**Table 1:** Prevalence of elephants with at least one lesion (active/inactive*), compared to prevalence of elephants with at least one active lesion by tourism camp in Thailand

Camp	#Elephants	Prev. (95 % CI^^^) elephants with at least one lesion (active/inactive)	Prev. (95 % CI^^^) elephants with at least one active lesion
**1**	13	69.2 (38.6 – 90.9)	30.8 (9.10 – 61.4)
**2**	8	25.0 (3.20 – 65.1)	0 (0 – 36.9^#^)
**3**	27	74.1 (53.7 – 88.9)	37.0 (19.4 – 57.6)
**4**	5	80.0 (28.4 – 99.5)	20.0 (0.5 – 71.6)
**5**	10	90.0 (55.5 – 99.7)	80.0 (44.4-97.5)
**6**	4	0 (0 – 60.2^#^)	0 (0 – 60.2^#^)
**7**	3	100 (29.2 – 100^#^)	100 (29.2 – 100^#^)
**8**	4	100 (39.8 – 100^#^)	75.0 (19.4 – 99.4)
**9**	26	92.3 (74.9 – 99.1)	84.6 (65.1 – 95.6)
**10**	9	100 (66.4 – 100^#^)	100 (66.4 – 100^#^)
**11**	4	100 (39.8 – 100^#^)	100 (39.8 – 100^#^)
**12**	5	100 (47.8 – 100^#^)	100 (47.8 – 100^#^)
**13**	8	87.5 (47.3 – 99.7)	75.0 (34.9 – 96.8)
**14**	18	100 (81.5 - 100^#^)	94.4 (72.7 – 99.9)
**15**	32	87.5 (71.0 – 96.5)	75.0 (56.6 – 88.5)
**16**	3	100 (29.2 – 100^#^)	66.7 (9.4 – 99.2)
**17**	6	50.0 (11.8 – 88.2)	16.7 (0.4 – 64.1)
**18**	9	88.9 (51.8 – 99.7)	66.7 (29.9 – 92.5)

*An active lesion is defined as a lesion categorized as rubbed/pink, raw, full depth ulcer, abscess (closed or draining), healing lesion, depigmented skin, or other. An inactive lesion is defined as a lesion categorized as either a healed lesion, or a callus

^^^Exact 95 % confidence interval

^#^One-sided, Exact 97.5 % confidence interval

**Table 2:** Summary of independent variables: working hours/day, break, saddle weight, contact material, age, and the number of elephants, by tourism camp in Thailand

Camp	#Elephants	Scheduled working hours/day	Break*	Saddle weight (kg)**	Contact material^^^	Average age in years [range] (# of elephants with known age)
**1**	13	10	Yes	Light	Shredded bark, carpet, blanket	28.1 [18 – 36] (13)
**2**	8	8	No	Heavy	Shredded bark, carpet, blanket	32.1 [17 – 47] (7)
**3**	27	8	No	Light	Shredded bark, carpet, blanket, rice sack	31.9 [28 – 35] (8)
**4**	5	6, 8, 10	No	Heavy	Shredded bark, blanket	47.0 [36 – 60] (5)
**5**	10	9	Yes	Light	Shredded bark, bark	44.1 [31 – 51] (9)
**6**	4	4	No	Light	Shredded bark	29.0 [12 – 40] (4)
**7**	3	5	No	Heavy	Rice sack	39.3 [37 – 41] (3)
**8**	4	7	Yes	Light	Shredded bark	33.0 [33] (1)
**9**	26	7	No	Heavy	Shredded bark, blanket	ND
**10**	9	7	No	Light	Shredded bark, blanket, rice sack	40.9 [31 – 52] (7)
**11**	4	4	No	Light	Rice sack	24.0 [14 – 40] (4)
**12**	5	7	No	Light	Rice sack	28.6 [15 – 45] (5)
**13**	8	6	No	Light	Rice sack	25.5 [17 – 35] (4)
**14**	18	4	Yes	Heavy	Carpet	33.8 [9 – 52] (18)
**15**	32	10	Yes	Heavy	Blanket	25.3 [7 – 45] (26)
**16**	3	2	Yes	Heavy	Shredded bark	39.0 [21 – 50] (3)
**17**	6	2	Yes	Heavy	Shredded bark	32.5 [15 – 59] (6)
**18**	9	2***, 7	1 yes , 8 no	Heavy	Shredded bark	33.1 [6 – 43] (9)

ND = No data

*Break is defined as a period of time when either the entire saddle was completely removed, or the girth strap was loosened

**Saddle weight is dichotomized into light (<10 kg) and heavy (>10 kg)

***The individual elephant that works 2 hours/day gets the break

^^^Each elephant had only one contact material, but the same material was not necessarily used for all animals within a camp

### Risk factor analysis

Initially, the multilevel model included random intercepts for both elephant and camp. However, the random intercept for elephant was removed because the variance component was negligible (i.e., < 1 × 10^−15^) and model fit, based on AIC and a likelihood-ratio test, was not improved by its inclusion. It should be noted that the neck region variable was removed from both univariable and multivariable analyses. There were so few lesions found in this region that it resulted in estimation problems for coefficients in our multilevel multivariable model.

### i) Univariable analysis

Based on univariable analysis, active lesions were more likely to be found in the back region in comparison to the other anatomical areas (i.e., girth, tail; Table 3). Increasing age was also identified by univariable analysis as being a risk factor for having an active lesion (Table 3). There appeared to be a significant quadratic relationship between working hours/day and the log odds of having an active lesion (Table 3). There was no significant difference in the prevalence of active lesions associated with the use of a heavy (>10 kg) or light (<10 kg) saddle, or with whether or not the elephants were provided a break (Table 3). We observed no significant differences between the use of the three contact materials (rice sacks, carpet, and blankets) compared to bark.

**Table 3:** Univariable multilevel* logistic regression models of factors associated with the presence of an active lesion on elephants from tourism camps in Thailand

Variable	Odds ratio	Lower 95 % CI	Upper 95 % CI	P value
**Body Area** (n = 194, m = 18)				
Girth	Ref			
Back	2.56	1.61	4.07	<0.0001
Tail	0.44	0.26	0.74	0.002
**Contact Material** (n = 163, m = 18)				
Bark	Ref			
Carpet	0.52	0.15	1.79	0.301
Blanket	0.78	0.30	2.02	0.612
Rice Sack	2.10	0.74	5.96	0.164
**Saddle Weight** (n = 191, m = 18)				
Light (<10 kg)	Ref			
Heavy (>10 kg)	0.65	0.21	2.03	0.458
Break** (n = 194 , m = 18)				
No	Ref			
Yes	0.54	0.18	1.67	0.286
**Age** (n = 133, m = 17)				
	1.03	1.002	1.05	0.034
**Working Hours/Day** (n = 194, m = 18)				
Working Hours/Day	3.79	1.41	10.18	0.008
Working Hours/Day Squared	0.90	0.83	0.97	0.009

Ref = Referent Category

n = number of elephants with data collected for the indicated variable (/194)

m = number of camps with data collected for the indicated variable (/18)

*Models include a random intercept for camp

**Break is defined as a period of time when either the girth strap was loosened, or the whole saddle was completely removed

### ii) Multivariable analysis

**Fig. 1:**
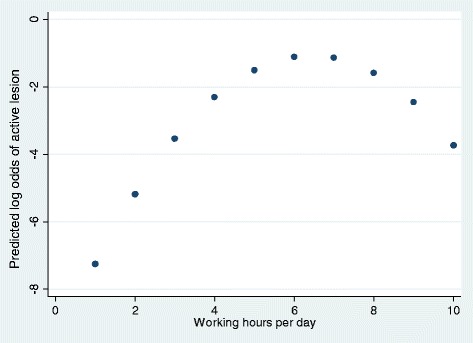
Relationship between the predicted log odds of an active lesion and hours worked per day. Predicted log odds of active lesion versus number of hours worked per day based on final multilevel multivariable logistic regression model for factors associated with active lesions on elephants from tourism camps in Thailand (all other variables held to referent or mean values)

In the final multilevel multivariable model, the following variables were included: body region, contact material, saddle weight, the provision of a break, age, and working hours/day and working hours/day squared. As observed in the univariable analysis, the back region was found to be at higher odds for having an active lesion compared to the girth and tail regions (Table 4). In comparison to each of the bark, carpet, and blanket materials, the use of a rice sack was found to be a significant risk factor for having an active lesion (Tables 4 and 5). Further comparisons between the bark, carpet, and blanket materials revealed no significant difference in the odds of having an active lesion (Table 5). Increasing age was found to be associated with increased odds of having an active lesion. A significant quadratic term was again observed for working hours/day. Initially the odds of having an active lesion increased with working hours/day; however this relationship peaked at 6–7 hours, followed by a decline (Fig. 1). After controlling for the other variables in the model, receiving a break significantly increased the odds of having an active lesion (Table 4). No significant difference was found between heavy and light saddles in association with the odds of having an active lesion. However, this variable was found to act as a confounder for both contact material and age, and was retained in the final model.

**Table 4:** Multivariable multilevel* logistic regression model of factors associated with the presence of an active lesion on 3 sites on elephants working in tourism camps in Thailand

Variable	Odds ratio	Lower 95 % CI	Upper 95 % CI	P value
**Body Area**				
Girth	Ref			
Back	2.92	1.55	5.50	0.001
Tail	0.46	0.23	0.92	0.028
**Contact Material**				
Bark	Ref			
Carpet	0.38	0.09	1.65	0.195
Blanket	0.63	0.13	2.96	0.558
Rice Sack	5.09	1.36	19.06	0.016
**Saddle Weight**				
Light (<10 kg)	Ref			
Heavy (>10 kg)	2.69	0.82	8.79	0.101
Break**				
No	Ref			
Yes	11.12	2.25	54.97	0.003
**Age**				
	1.03	1.002	1.06	0.036
**Working Hours/Day**				
Working Hours/Day	23.74	5.17	109.03	<0.0001
Working Hours/Day Squared	0.78	0.69	0.89	<0.0001

Variance = 0.48 (95 % CI 0.08 – 2.73)

*Model includes a random intercept for camp

**Break is defined as a period of time when either the girth strap was loosened, or the whole saddle was completely removed

**Table 5:** Summary of multilevel* multivariable logistic regression comparisons between the four padding materials in contact with the skin: rice sack, bark, blanket, and carpet, used on elephants working in tourism camps in Thailand

Comparison	Odds ratio	Lower 95 % CI	Upper 95 % CI	P value
Rice Sack – Blanket	8.09	1.16	56.45	0.035
Rice Sack – Carpet	13.52	2.22	82.35	0.005
Rice Sack – Bark	5.09	1.36	19.06	0.016
Blanket – Bark	0.63	0.13	2.96	0.558
Carpet – Bark	0.37	0.09	1.65	0.195
Carpet – Blanket	0.60	0.09	3.90	0.591

Note: Odds ratios are calculated using the second listed variable as the referent category

*Model used for comparisons includes a random intercept for camp

An intercept only analysis revealed that 20 % of the model variance was explained at the camp level. In the final multilevel multivariable analysis, we found 13 % of the variance in the outcome was accounted for at the camp level. We observed no outliers based on examination of the Pearson residuals. Based on visual examination of the BLUPs, we found that model assumptions concerning normality and homogeneity of variance were met.

## Discussion

The prevalence and associated risk factors for skin lesions were examined using data from 194 working elephants from 18 Thailand tourism camps. Descriptive analyses revealed a high prevalence of active lesions among these elephants especially in the back region. Risk factors associated with increased odds of having an active lesion were identified as: body region (i.e., back region), the use of rice sacks as padding material in contact with the skin, increasing age of the elephant, the provision of a break, and longer working days.

A high prevalence of active lesions associated with working conditions was found, as 64.4 % of these elephants had at least one active lesion, and 82.5 % had at least one lesion (active or inactive). This finding is comparable to the prevalence of pack wounds found in various studies on working equids (77.5 % [15], 70.9 % [16], 72.1 % [17], 54.4 % [18]). Interestingly, virtually none of the model variance was explained at the elephant level, suggesting lesions among sites on an elephant occur relatively independently of each other. Approximately 13 % of the model variance was explained at the camp level, even after accounting for several camp level variables. These variables included working hours/day, the provision of a break, and saddle weight. This suggests that camp level management has a moderate impact on elephant welfare associated with external injuries, potentially indicating an area for more detailed investigation in future studies.

The back region appears to be a high risk area for the presence of an active lesion relative to the other anatomical sites, followed by the girth region. These findings correspond to the results of various studies regarding working donkeys and mules. Sells et al. identified the withers and shoulder as the regions of highest lesion prevalence, at 40 % and 31 %, respectively [18]. Biffa and Woldemeskel saw the highest lesion prevalence on the withers at 18.6 % [17]. Pearson et al. documented the back region to be the area of highest lesion prevalence at 90-93 %, and the neck region as a lower risk area at 4-10 % [14]. Pritchard et al. saw lesions on the head, neck, ribs, flank and tail base in less than 10 % of equids studied, and observed the highest prevalence of lesions in mules on the breast/shoulder, withers and girth regions (22.5, 21.3 and 28.4 %, respectively) [16]. The results of our study, along with these comparable equid results, suggest a primary area for intervention. Future studies and current management/veterinary care regarding working elephants should focus efforts primarily on the back region and secondarily on the girth region, as opposed to the neck and tail, where lesions were relatively rare.

Padding material in contact with the skin was a significant factor for the prevalence of active lesions. In particular, rice sacks significantly increased the odds of having an active lesion relative to all other padding materials. Avoiding their use in favour of bark, carpet, or blankets would be beneficial in terms of decreasing the odds of having an active lesion. Bark, carpet and blankets were not found to be significantly different from one another; therefore, at this time there are no recommendations for a potential standard material.

Increasing age had an expected association with increasing odds of having an active lesion. Although an odds ratio of 1.03 is relatively small, if we take into account that these are long lived animals, the odds of an active lesion increases 1.56 times for every 15 year increase in age. Due to the nature of a cross-sectional study, there is an issue concerning prevalence vs. incidence of active lesions on older animals. The study design did not allow us to discern whether older animals were more likely to have active lesions due to increased susceptibility, decreased healing times, or some combination of both. Regardless, at this time, we recommend that a higher level of monitoring/attention be given to older elephants by their owners or caretakers. It is important to note that due to the inclusion of the age variable, which had the largest amount of missing data (31.7 %), the number of elephants included in the final model decreased from 162 to 113. However, the direction (i.e., sparing or risk factor) of the odds ratios of the other variables remained the same; only the magnitude of these effects was altered. As we thought age was an important biological variable that showed statistical significance, we decided to include it in the final model despite the loss of some observations for the multivariable analysis.

Surprisingly, the provision of a break was found to be a risk factor associated with increased odds of having an active lesion. It is possible that camps that offer more breaks may be doing so in response to a current or previous problem with lesions. But again, as a result of the limitations of a cross-sectional study, the incidence of active lesions as a result of breaks could not be investigated directly. Control trials or cohort studies should be conducted to investigate the effect of breaks on the incidence of lesions along with more specific descriptions concerning their timing (i.e., number, length, and scheduling) and the conditions associated with these breaks. For instance, the loosening of the girth strap during a break may cause more movement of the saddle equipment and, therefore, more abrasions.

The association between the odds of having an active lesion and working hours/day had a quadratic relationship. The predicted log odds of an active lesion were observed to initially increase with increasing working hours/day, followed by a peak at 6–7 hours and subsequent decline. This drop was an unexpected finding, but may be related to the “healthy worker bias”. For instance, more resilient elephants may be selected more frequently to work more hours, or those elephants that sustain too many lesions may have decreased numbers of working hours, or be removed from the camp. This could result in a selection bias and an impression of a healthier population of elephants working longer hours, which may explain the quadratic relationship identified. Sells et al. found a similar result in their study analysing working equids in Morocco, where increased frequency of work/week was found to be associated with a decreased risk of sustaining a pack wound [18]. They also identified a healthy worker selection bias as a possible explanation for these findings [18]. Due to the nature of the present study, the prevalence of skin lesions was observed only in animals currently working in the camp, and data regarding total working hours/day were gathered only for current management. Therefore, to control for this potential bias, future studies analysing the role of working hours should examine all elephants that have been used by the camp, and take information on any changes in working hours among the elephants. It may be prudent at this time to recommend limiting work to <6 hours/day since the odds of observing an active lesion was highest at this time point.

Although the weight of the saddle was found to be a confounding variable for the present study, a significant difference between heavy (>10 kg) and light (<10 kg) saddles was not found to be associated with an increased odds of having an active lesion. However, multiple studies investigating similar factors among working equids have implicated overloading as a major causal factor for skin lesions [14, 17, 18]. Kontogeorgopoulos also cautions that although these elephants can carry up to 300 kg, it is recommended they carry no more than 200 kg [4]. Therefore, the total weight that elephants carry (i.e., saddle + tourists) should be taken into consideration. It is possible that relative to the individuals being carried, the weight of the saddle is not significant. It is also possible that the lack of variability in the weight variable within camps may have impacted our ability to find an effect of weight on lesion outcome. It is also important to note that by dichotomizing the weight of the saddles due to limitations in their assessment in the field, a great deal of information was lost that could have been identified if modelled as a continuous variable.

## Conclusions

In conclusion, lesions related to working conditions were found to be highly prevalent among the tourism elephants studied. These lesions were found to be associated with risk factors including the use of rice sacks as padding material in contact with the skin, longer working days (to an extent), and the provision of a break. The back region was identified as being at an increased risk of having an active lesion, followed by the girth region. While the cross-sectional nature of the study posed several inferential limitations, at this time we recommend the following until longitudinal studies can be conducted: increased monitoring of older elephants, increased monitoring of the back region of elephants of all ages, working hours limited to less than 6 hours per day, and the avoidance of rice sacks as padding material in contact with the skin.

## Additional file

Additional file 1:
**Elephant Observation Sheet.**

## References

[1] Lair RC (1997). Gone astray: the care and management of the Asian elephant in domesticity.

[2] Lohanan R, Baker I, Kashio M (2002). The elephant situation in Thailand and a plea for co-operation. Giants on our hands: Proceedings of the international workshop on the domesticated Asian elephant: February 5–10, 2001; Bangkok, Thailand.

[3] Godfrey A, Kongmuang C (2009). Distribution, demography and basic husbandry of the Asian elephant in the tourism industry in Northern Thailand. Gajah.

[4] Kontogeorgopoulos N (2009). The role of tourism in elephant welfare in Northern Thailand. J Tourism.

[5] Kontogeorgopoulos N (2009). Wildlife tourism in semi-captive settings: a case study of elephant camps in Northern Thailand. Current Issues Tourism.

[6] Chatkupt TT, Sollod AE (1999). Elephants in Thailand: determinants of health and welfare in working populations. J Appl Anim Welf Sci.

[7] Tipprasert P, Baker I, Kashio M (2002). Elephants and ecotourism in Thailand. Giants on our hands: Proceedings of the international workshop on the domesticated Asian elephant: February 5–10, 2001; Bangkok, Thailand.

[8] Hatt JM, Clauss M (2006). Feeding Asian and African elephants. Int Zoo Yb.

[9] Wang L, Lin L, He Q, Zhang J, Zhang L (2007). Analysis of nutrient components of food for Asian Elephants in the wild and in captivity. Front Biol China.

[10] Duffy R, Moore L (2011). Global regulations and local practices: the politics and governance of animal welfare in elephant tourism. J Sustain Tour.

[11] Mikota SK, Maslow JN (2011). Tuberculosis at the human-animal interface: an emerging disease of elephants. Tuberculosis.

[12] Vanitha V, Thiyagesan K, Baskaran N (2011). Social life of captive Asian elephants (*Elephas maximus*) in Southern India: implications for elephant welfare. J Appl Anim Welf Sci.

[13] Khawnual P, Clarke B, Baker I, Kashio M (2002). General care and reproductive management of pregnant and infant elephants at the Ayutthaya Elephant Camp. Giants on our hands: Proceedings of the international workshop on the domesticated Asian elephant: February 5–10, 2001; Bangkok, Thailand.

[14] Pearson RA, Alemayehu M, Tesfaje A, Allan EF, Smith DG, Asfaw M (2001). Use and management of donkeys in peri-urban areas of Ethiopia.

[15] Curran MM, Feseha G, Smith DG (2005). The impact of access to animal health services on donkey health and livelihoods in Ethiopia. Trop Anim Health Prod.

[16] Pritchard JC, Lindberg AC, Main DCJ, Whay HR (2005). Assessment of the welfare of working horses, mules and donkeys, using health and behaviour parameters. Prev Vet Med.

[17] Biffa D, Woldemeskel M (2006). Causes and factors associated with occurrence of external injuries in working equids in Ethiopia. Int J Appl Res Vet Med.

[18] Sells PD, Pinchbeck G, Mezzane H, Ibourki J, Crane M (2010). Pack wounds of donkeys and mules in the Northern High Atlas and lowlands of Morocco. Equine Vet J.

