# Supplementary material for: Risk factors for saddle-related skin lesions on elephants used in the tourism industry in Thailand
Source: BMC Vet Res. 2015 May 19;11:117. doi: 10.1186/s12917-015-0438-1 (PMC4437249; doi:10.1186/s12917-015-0438-1)
Supplement: Additional file 1: — Elephant Observation Sheet. [file 12917_2015_438_MOESM1_ESM.docx]

**Additional file 1**

**Elephant Observation Sheet**

**Date:**

**Microchip number:**

**Sex: M F**

**Age:**

**Mahout:**

**Owner:**

**How long has elephant had this harness?**

**How many hours a day does this elephant work?**

**Does the elephant receive a break? If so, how long and is the saddle removed or left on?**

**Saddle materials:**

**Saddle design:**

**Lesions seen on elephant:**

**LEGEND**

| 1 | RUBBED/PINK AREA OF SKIN |
| --- | --- |
| 2 | RAW |
| 3 | FULL DEPTH ULCERATED SKIN |
| 4 | ABSCESS (CLOSED) |
| 5 | PUS DRAINING ABSCESS |
| 6 * | HEALING LESION |
| 7 * | HEALED LESION |
| 8 * | CALLUS |

| **Saddle site** | # lesions | Type (side L/R) | Size (cm) | Score (1-8) |
| --- | --- | --- | --- | --- |
| Girth (GL/GR) |  | worst |  |  |
|  |  | mild |  |  |
| Back (BL/BR) |  | worst |  |  |
|  |  | mild |  |  |
| Tail (TL/TR) |  | worst |  |  |
|  |  | mild |  |  |
| Neck (TL/TR) |  | worst |  |  |
|  |  | mild |  |  |
